# The Feasibility and Relevance of Collecting Adolescent Health Indicators in Humanitarian Settings: Results From the West Bank

**DOI:** 10.1111/hex.70324

**Published:** 2025-06-13

**Authors:** Aisha Shalash, Niveen Abu Rmeileh, Dervla Kelly, Khalifa Elmusharaf

**Affiliations:** ^1^ Institute of Community and Public Health Birzeit University West Bank Occupied Palestinian Territory; ^2^ School of Medicine University of Limerick Limerick Ireland; ^3^ College of Health Sciences Qatar University Doha Qatar; ^4^ School of Public Health University of Birmingham Dubai United Arab Emirates

**Keywords:** adolescent health, feasibility, humanitarian settings, indicators, relevant, West Bank

## Abstract

**Background:**

There is a critical concern for adolescent health in the Eastern Mediterranean region especially during humanitarian crises. This study emphasises not only the need for measuring key health indicators but also how to collect the information effectively, particularly in the West Bank. These efforts will guide evidence‐based policies and interventions for this population.

**Setting and Participants:**

This mixed‐methods study aimed to determine the perceived relevance and feasibility of collecting a set of adolescent health indicators in the West Bank. Stakeholders filled out a questionnaire and participated in a workshop where they ranked 50 indicators based on context specificity, usefulness, feasibility, timeliness and resource availability. The researchers analysed the data and categorised the indicators as feasible and relevant, feasible but irrelevant, not feasible but relevant, or not feasible and irrelevant.

**Results:**

The results of the study show that 22 out of the 50 adolescent health indicators were perceived as both feasible and relevant to be collected in the West Bank. These indicators cover a range of content areas, including determinants of health, mortality, morbidity, health behaviour and risks, policies, systems, interventions, and mental health and well‐being. However, some indicators were deemed relevant but not feasible due to cultural norms, stigma and wording issues, while others were considered not relevant and not feasible, including certain indicators related to alcohol use and unavailable practices and services. Despite the challenges, stakeholders acknowledged the importance of creating a national list of adolescent health indicators to guide evidence‐based programmes and policies, emphasising the need for governmental support and infrastructure for data collection.

**Conclusion:**

Cultural norms, stigma and wording issues emerged as significant challenges in data collection. Recommendations include implementing comprehensive sexual education, improving mental health indicator wording and investing in resources to support data collection and measurement. These findings will help guide evidence‐based programmes and policies for adolescent health in the region. The establishment of a national list of adolescent health indicators services is a milestone for standardising data collection, guiding health programme planning and fostering accountability. Overall, this study's findings will significantly improve understanding, interventions and prioritisation of adolescent health in the region, potentially influencing similar contexts worldwide.

**Patient or Public Contribution:**

Our study could not have been done without the involvement of service providers. Major adolescent service providers were provided with a list of indicators and asked to rank their feasibility and relevance. The results of their rankings were then shared in a workshop. A presentation was given on the ranking of the stakeholders, and they were given a chance to discuss any agreements or concerns with the tentative list of indicators.

## 1 Introduction

The Eastern Mediterranean region is the home of 22 countries. Over half of these countries face a humanitarian crisis [1]. In this context, numerous humanitarian organisations operating in the region have led to a lack of government control over the selection and implementation of programmes and interventions [2]. During this time, adolescents' well‐being becomes a critical concern. Adolescents (aged 10–19) are in a crucial stage of development and represent a significant portion of the population in humanitarian settings. Yet, programming and interventions sometimes overlook their health needs [3]. Due to their unique physical and emotional development, adolescents require specialised attention different from that of adults and children [4]. In times of humanitarian crises, some male adolescents are forced to take on the role of head of household. In contrast, female adolescents are at a higher risk of early marriage and gender‐based violence [5]. This puts them in more vulnerable situations, which, combined with other factors, contribute to some of the leading causes of death among adolescents in the Eastern Mediterranean Region [6]. These causes include collective violence, road injuries, congenital anomalies, lower respiratory infection, tuberculosis, iron‐deficiency anaemia, self‐harm, maternal causes, interpersonal violence and major depressive disorders [7].

Health indicators are collected using different health surveys, census data and population‐based surveys such as the Demographic Health Survey and the Multiple Indicator Cluster Survey [8]. During humanitarian settings, this can be challenging because of safety and the population's rapidly changing needs. Rapid needs assessments are often relied upon in humanitarian settings and are not widely shared frequently; therefore, there is a lack of standardisation and measurability in these assessments, and they cannot be used to inform policies and programmes [9]. In a previous study done by the researchers, it was found that adolescent health indicators are collected in humanitarian settings, using population‐based surveys and are often out of data and not inclusive of both males and females [10]. It is crucial to monitor key adolescent health indicators to strengthen an already weakened health system [11].

It is important, before data collection, to have an understanding of the relevance and feasibility of adolescent health indicators to ensure efficient resource allocation, cultural acceptability and alignment with local priorities. For example, indicators related to sensitive topics require careful consideration to prevent resistance and underreporting. Involving local stakeholders in the future helps promote acceptability and allows for their inputs to ensure the selected indicators align with their values and needs [12]. This need is particularly evident in humanitarian settings, where additional efforts are required to collect and provide accurate, up‐to‐date data to monitor and evaluate humanitarian actions. However, responsibilities and accountabilities for this study are often unclear [13]. While evidence from non‐humanitarian settings may apply to different populations across countries, certain conditions, such as malnutrition, are unique to humanitarian settings [14]. Health indicators are essential for tracking the progress of national health service delivery goals [15]. Unfortunately, only 6% of the Sustainable Development Goal indicators focus explicitly on adolescent health [16].

Considering the surge of non‐governmental organisations in the West Bank amid the ongoing humanitarian crisis and the lack of governmental control over available programmes, it is vital to invest time and effort in measuring key indicators. Evidence‐based programmes and policies are necessary in this regard. In humanitarian settings like the West Bank, collecting health indicators is hindered by cultural norms and resource limitations, making it important to first identify indicators that are both contextually relevant and feasibly collectable [17]. The Palestinian Coalition of Adolescent Health Launching Event took place in August 2019, bringing together various governmental and non‐governmental stakeholders to address the current needs, concerns and perceptions of adolescents and youth in the occupied Palestinian territory. In August 2022, the strategic plan for adolescent and youth health was launched, outlining the current needs of adolescents in the country and establishing the adolescent and youth department within the Ministry of Health. However, the strategy did not specify the key indicators necessary to measure the progress of the implemented programmes or policies.

Recognising the need to address the specific challenges and needs of adolescents, the WHO and United Nations partner agencies established the Global Action for Measurement of Adolescent Health (GAMA) Advisory Group (AG) [18]. Since 2018, the GAMA AG has defined a set of core measurement areas for adolescent health, mapped existing indicators linked to these areas and selected a draft list of priority indicators for adolescent health measurement [19, 20, 21]. Using this list to determine which indicators are relevant and feasible, this study provides the exploratory research for evidence‐based interventions and a framework for future data collection efforts in similar humanitarian settings [22]. Involving different stakeholders and prioritising a country's adolescent health needs is essential in focusing on adolescent health interventions. This process considers the severity of the country's issues, the availability of interventions, the most affected groups or populations and the feasibility of executing interventions. Collecting data on significant and feasible indicators is crucial [23]. This study aims to determine the perceived relevance and feasibility of collecting a set of adolescent health indicators in the West Bank.

## 2 Methods

The study employed a structured approach to assess the perceived relevance and feasibility of adolescent health indicators in the West Bank, incorporating stakeholder engagement through a multistep process. Initially, stakeholders were invited to complete a questionnaire designed to evaluate a comprehensive set of indicators based on predefined criteria. Following this, a workshop was convened to critically analyse and validate the findings, fostering collaborative discussion and consensus‐building among participants.

The list of indicators was based on adolescent health indicators suggested by the World Health Organization GAMA (WHO GAMA) [12]. Each indicator was translated from English to Arabic and back to English by the researchers to ensure the meaning was preserved, and both languages were included on the ranking sheet provided to stakeholders. The list of indicators and their translations can be found in Annex 1.

Purposive sampling was used to invite the stakeholders to participate. Stakeholder mapping was done in a previous research, which allowed the researchers to have a cumulative list of adolescent health organisations. Participants were directly identified and invited if they were recognised for their involvement in organisations serving adolescent health or contributing to their organisation's Health Information Systems (HIS). Selection criteria focused on their expertise in HIS or their experience working with adolescents. Stakeholders from the Ministry of Health, the Ministry of Education, International and Local organisations, and academic institutes were approached in person or by email to participate in ranking adolescent health indicators. They were informed that the ranking of indicators will allow for the collection of relevant and feasible indicators to establish evidence‐based programmes and policies for adolescents to improve adolescent health outcomes. If the participant agreed, a hard copy was physically given to the participant, and a soft copy was then subsequently emailed. Up to three follow‐up emails were sent to each participant.

The stakeholders were asked to rate each of the 50 indicators on a 5‐point Likert‐type scale (5 = strongly agree, 4 = agree, 3 = unsure, 2 = disagree and 1 = strongly disagree) on five criteria. These criteria were (1) Context‐specific, (2) Feasibly measurable, (3) Timely collectable, (4) Financial and Human Resources available and (5) Useful and Important. These criteria were based on expert consultation and the literature of the criteria of quality indicators [24]. Table 1 provides a detailed description of how the indicators were assessed.

**Table 1:** The five criteria for assessing the 50 indicators.

Main criteria	Ranking
Relevance	
1. Context‐specific	5—Strongly agree: The indicator is highly specific to the Palestinian context 4—Agree: The indicator is specific to the Palestinian context 3—Unsure: Unsure whether the indicator is specific to the Palestinian context 2—Disagree: The indicator is not specific to the Palestinian context 1—Strongly disagree: The indicator is not highly specific to the Palestinian context
2. Useful and/or important	5—Strongly agree: The indicator is very important for the Palestinian context 4—Agree: The indicator is important for the Palestinian context 3—Unsure: Unsure whether the indicator is important for the Palestinian context 2—Disagree: The indicator is not important for the Palestinian context 1—Strongly disagree: The indicator is not very important for the Palestinian context
Feasibility	
3. Feasibly measurable	5—Strongly agree: The data for the indicator can easily be collected in Palestine 4—Agree: The data for the indicator can be collected in Palestine 3—Unsure: Unsure if the data for the indicator can be collected in Palestine 2—Disagree: The data for the indicator cannot be collected in Palestine 1—Strongly disagree: The data for the indicator cannot be easily collected in Palestine
4. Timely measurable	5—Strongly agree: The indicator can easily be measured regularly in Palestine 4—Agree: The indicator can be measured regularly in Palestine 3—Unsure: Unsure whether the indicator can be measured regularly in Palestine 2—Disagree: The indicator cannot be measured regularly in Palestine 1—Strongly disagree: The indicator cannot be measured regularly in Palestine
5. Financial and human resources availability	5—Strongly agree: The financial and human resources are readily available to measure this indicator in Palestine 4—Agree: The financial and human resources are available to measure this indicator in Palestine 3—Unsure: Unsure whether the financial and human resources are readily available to measure this indicator in Palestine 2—Disagree: The financial and human resources are not available to measure this indicator in Palestine 1—Strongly disagree: The financial and human resources are not readily available to measure this indicator in Palestine

After each of the ratings, each participant could put in free text comments considering why they rated each indicator or any other comments they wanted to share. They either physically returned the questionnaire with the rating or emailed their selections. Each participant was given 2–3 weeks to return their ratings to the researcher. Reminders were emailed, or participants were called after the requested submission date to ensure their participation. A workshop was held where all stakeholders were invited to participate in the sharing of the preliminary results. A presentation was given on the ranking of the stakeholders, and they were given a chance to discuss any agreements or concerns with the tentative list of indicators.

## 3 Data Analysis

Using the five criteria listed above, the researchers grouped the indicators into two groups: relevancy and feasibility. Relevancy was determined for each participant by the mean of the context‐specific, useful and important responses, and feasibility was determined by the mean score of participants of feasibly measurable, timely collectable, and financial and human resource availability. Then, for each indicator, the meaning of all participants was taken for both relevancy and feasibility. To analyse the results, the following interpretation of values was used: 4.50–5.00 Strongly agree; 3.5–4.49 Agree; 2.51–3.49 Neutral; 1.50–2.49 Slightly agree and 1.00–1.49 Disagree.

An average of 3.5 or higher for each group was considered feasible and relevant because we were interested in the indicators rated Strongly agree (4.50–5.00) and Agree (4: 3.5–4.49). This allows each of the 50 indicators to fall into one of four categories, respectively: feasible and relevant ( < 3.49 and < 3.49), feasible and irrelevant ( < 3.49 and > 3.49), not feasible and relevant ( > 3.49 and < 3.49), and not feasible and irrelevant ( > 3.49 and > 3.49). Descriptive statistical analysis, including the calculation of mean scores and categorisation analysis, was done using Microsoft Excel. All comments left by the participants were compiled and discussed in the workshop that was later held to discuss the preliminary results.

The workshop was audio‐recorded with the informed consent of all participants, ensuring that the recording would only be shared with the research team and their contributions would stay anonymous. After the session, the recording was transcribed by a research assistant. An inductive reasoning approach was employed using MAXQDA software to analyse the data. This method allowed themes to emerge from the discussion, reflecting on the participants' insights without preconceived themes.

## 4 Results

Sixteen of 22 stakeholders (84.2% response rate) returned their questionnaires. All 22 stakeholders were invited to the workshop. Fourteen out of the 16 stakeholders attended the discussion of indicators at the workshop conducted. Stakeholders perceived that out of the 50, 22 were feasible and relevant to be collected in the West Bank. Table 2 describes the 16 stakeholders based on organisational affiliation, experience in HIS or adolescent health and who attended the workshop. Below you will find the different categorisations of indicators.

**Table 2:** Characteristics of participants: experience and organisation type.

Identification (ID) number	Organisation type	Years of experience	Present at workshop
1	Governmental	28 years	Yes
2	Local NGO	12 years	Yes
3	Local NGO	14 years	Yes
4	Local NGO	33 years	No
5	International NGO	20 years	Yes
6	Local NGO	14 years	Yes
7	Governmental	15 years	Yes
8	Governmental	22 years	Yes
9	Governmental	17 years	Yes
10	Governmental	14 years	Yes
11	Governmental	27 years	Yes
12	International NGO	13 years	Yes
13	International NGO	20 years	No
14	Academic Institute	16 years	Yes
15	International NGO	15 years	Yes
16	Local NGO	12 years	Yes

## 5 Categorisation of Indicators

The indicators were distributed into eight different content areas: determinants of health, population, mortality, morbidity, sexual and reproductive health, health behaviour and risks, violence and injuries, policies, systems and interventions, and mental health and well‐being. No indicator was found feasible and not relevant. There are 11 indicators dedicated to sexual and reproductive health; four were found relevant, and none were found to be feasible. The most feasible indicators are in the categorisation of health behaviour and risks, where out of nine indicators, seven were relevant and six were feasible. One mental health indicator and one violence and injury indicator were found to be both relevant and feasible. The distribution of the indicators and categorisation can be found in Table 3. There were 22 (44%) that were relevant and feasible, no indicators that were not relevant and feasible, 17 (34%) that were relevant and not feasible, and 11 (22%) that were not relevant and not feasible. No content area was found to be fully relevant and feasible.

**Table 3:** Categorisation of indicators based on content area, relevancy and feasibility.

Content area	Number of Indicators	Relevant	Feasible
Determinants of health	6	4	4
Population, mortality and morbidity	4	4	3
Sexual and reproductive health	11	4	0
Health behaviour and risks	9	7	6
Violence and injuries	5	5	1
Policies, programmes and laws	4	4	3
Systems, performances and Interventions	5	5	4
Mental health and well‐being	6	6	1
Total	50	39 (78%)	22 (44%)

## 6 Perceived Relevant and Feasible List of Indicators

Twenty‐two indicators were identified to be relevant and feasible. The scores for relevance were much higher than for feasibility. The feasibility scores were borderline (e.g., 3.5) for several of the indicators, such as living with food insecurity, thinness, physical activity, fruit and vegetable servings, physical violence, reporting of suicide and the existence of a functioning national programme. This shows some uncertainty in measuring these indicators and the resources available.

Living with food insecurity was the only indicator to receive a score lower than 4 in relevancy. Table 4 gives a complete description of the included indicators. While there were no quotes from the workshop to explain why stakeholders found these indicators relevant and feasible, their inclusion in the study suggests that they align with their priorities, and the current health system has existing resources for collection. Stakeholders were more interested in discussing why some were not feasible and why some indicators need to be included in the original list of indicators.

**Table 4:** Indicators that were considered both relevant and feasible.

Name of indicator	Relevance	Feasibility
Determinants of health (education and income level)		
1. Percentage of adolescents (10–19 years) not in education, employment or training, by age group (10–14 and 15–19 years) and sex	4.5	4.2
2. Percentage of adolescents completing primary, lower secondary and upper secondary school, by level and sex	4.8	4.4
3. Percentage of adolescents (10–19 years) living below the national poverty line, by age group (10–14 and 15–19 years) and sex	4.4	3.8
4. Percentage of adolescents (10–19 years) living with moderate or severe food insecurity in the population, based on the Food Insecurity Experience Scale (FIES), by age group (10–14 and 15–19 years) and sex	3.9	3.5
Demographics, mortality and morbidity		
5. Percentage of the total population that are adolescents (10–19 years), by age category (10–14 and 15–19 years) and sex	4.7	4.5
6. Adolescent (10–19 years) mortality rate by age group (10–14 and 15–19 years) and sex	4.7	4.5
7. Adolescent (10–19 years) mortality rate by specified causes of death, age group (10–14 and 15–19 years) and sex	4.7	4.4
Health behaviour and risks		
8. Prevalence of overweight and obesity among adolescents (10–19 years) by weight status (overweight and obese), age group (10–14 and 15–19 years) and sex	4.9	3.9
9. Prevalence of thinness among adolescents (10–19 years), by age group (10–14 and 15–19 years) and sex	4.5	3.5
10. Prevalence of current (past 30 days) use of tobacco products among adolescents (10–19 years) by age group (10–14 and 15–19 years), sex and type of tobacco used	4.8	3.7
11. Percentage of adolescents (10–19 years) who consume at least five servings of fruit and vegetables daily, by age group (10–14 and 15–19 years) and sex	4.4	3.5
12. Percentage of adolescents (10–19 years) who have accumulated an average of at least 60 min per day of moderate‐vigorous physical activity in the previous week, by age group (10–14 and 15–19 years) and sex	4.5	3.5
13. Percentage of adolescents (10–19 years) who usually drank sugar‐sweetened beverages once per day or more during the past 30 days, by age group (10–14 and 15–19 years) and sex	4.3	3.5
Policies, programmes and laws		
14. Existence of a functional adolescent (10–19 years) health programme with coverage at the national level	4.4	3.5
15. Existence of national standards for the delivery of health services to adolescents (10–19 years)	4.4	3.6
16. Existence of a legal age limit for married and unmarried adolescents (10–19 years) to provide consent, without spousal/parental/legal guardian consent, for specified adolescent health services, by marital status and type of service	4.1	3.8
Systems, performances and interventions		
17. Percentage of adolescents (10–19 years) using specified health services in the public or private sector within the past 12 months, by sector, age group (10–14 and 15–19 years) and sex	4.4	3.7
18. Existence of age‐ and sex‐disaggregated health data for adolescents (10–19 years) in the national health information system	4.8	4.0
19. Existence of a nationally defined minimum package of school‐based health and nutrition services based on local health priorities	4.6	4.1
20. Percentage of live births to female adolescents (10–19 years) attended by skilled health personnel, by age group (10–14 and 15–19 years)	4.7	4.4
Violence and injury		
21. Percentage of adolescents (10–19 years) involved in physical violence in the past 12 months, by type of involvement (victim, perpetrator and both), age group (10–14 and 15–19 years), sex, perpetrator (parents/caregivers, teachers, intimate partners and peers)	4.4	3.5
Mental health and well‐being		
22. Percentage of adolescents (10–19 years) reporting a suicide attempt in the past 12 months, by age group (10–14 and 15–19 years) and sex	4.4	3.5

## 7 Relevant but Not Feasible Indicators

Stakeholders ranked 17 out of 50 indicators as what they perceived were relevant but not feasibly collectible in the country. These were mainly due to cultural norms, stigma associated with indicator, and wording of indicators.

### 7.1 Cultural Norms

The indicators describing providing sex education, STIs and menstruation awareness were found to be relevant and not feasible due to some stakeholders expressing the lack of social acceptability amongst some parents and teachers. A stakeholder described that when reproductive health was described in a science book in school, a teacher told the students to read about it at home.

When a teacher gets to the reproductive health section of the science book, they quickly skip over it and tell the students to read about it at home. They are too shy to discuss it in class.

There is also the notion that if you discuss sexuality, then you are promoting it.

When you open their eyes (the students) to sex, contraception, STIs … it is like telling them that it is okay to go out and have sex, as long you do it safely.

### 7.2 Stigma

Reporting suicidal thoughts was also seen as a feasibility challenge. Suicide is considered religiously unacceptable, so adolescents are often afraid to express these thoughts if they exist. Workshop participants highlighted that violence and injury indicators involving sexual abuse are unfeasible due to the stigma that comes with sexual abuse. They emphasised that in many close‐knit communities, sexual abuse goes unreported due to the fear of being ostracised from the community. Similarly, participants noted that bullying often goes unreported due to fears of inadequacy and being outcasted for reporting. These would not be feasible to collect because we would not have the data to explore the indicators. This suggests the need for culturally sensitive approaches to data collection and support systems that build trust and reduce stigma.

## 8 Wording of Indicators

Feasibility was also a challenge among the mental health indicators. Some stakeholders voiced their concern about the wording of the indicators being misleading and not representative of mental and psychosocial support indicators. They felt that the indicators needed to be better described to be feasible. Situational factors should also be considered in the definition.

What does depression mean? It has different meanings and symptoms in different cultures and can label and stigmatize people.

The political unrest and chronic humanitarian crisis play a role in the mental health of adolescents in the country, making it hard to label someone as having depression or feeling depressed. This reflects the broader challenge of applying globally recognised mental health terms in contexts where chronic humanitarian settings shape adolescents' emotional experiences, where they could be experiencing depression because of the current humanitarian situation.

When we think depression, we think they are mentally ill. However, they could be experiencing depression because of the current humanitarian situation in the country.

Some stakeholders saw the wording of psychoactive drug use as a problem. Psychoactive drug use can refer to energy drinks, for example, caffeine intake, as well as the use of illegal substances. It was found that the indicator needed to be clearer if illegal substances were being described here. This shows the importance of developing globally comparable indicators and ensuring their relevance and interpretability in local contexts. Table 5 describes the indicators found to be relevant but not feasible and the reason for their exclusion.

**Table 5:** Description of indicators found to be relevant but not feasible and the reason for exclusion.

Name of indicator	Relevance	Feasibility	Reason for exclusion
Demographics, mortality and morbidity			
1. Prevalence of anaemia among adolescents (10–19 years) by age category (10–14 and 15–19 years) and sex	4.6	3.3	Hard to collect
Sexual and reproductive health			
2. Adolescent (10–19 years) fertility rate by age group (10–14 and 15–19 years)	3.9	3.1	
3. Percentage of female adolescents (10–19 years) who were aware of menstruation before menarche by age group (10–14 and 15–19 years)	4.4	3.4	Cultural norms
4. Number of new adolescents (10–19 years) HIV infections per 1000 uninfected adolescent population, by age group (10–14 and 15–19 years) and sex	3.6	3.3	Cultural norms
5. The incidence rate of sexually transmitted infections (STIs) among adolescents (10–19 years) by age group (10–14 and 15–19 years) and sex	3.5	2.8	Cultural norms
Health behaviour and risks			
6. Past 12‐month prevalence of psychoactive drug use among adolescents (10–19 years), by age group (10–14 and 15–19 years), sex and type of substances	4.1	2.4	Hard to collect
Policies, programmes and laws			
7. Existence of national policy exempting adolescents (10–19 years) from user fees for specified health services in the public sector by type of service	4.0	3.1	Resource allocation
Systems, performances and interventions			
8. Percentage of schools that provided life skills‐based HIV and sexuality education within the previous academic year	3.8	3.4	Cultural norms
Violence and injury			
9. Incidence rate of specified types of injuries among adolescents (10–19 years) and by age category (10–14 and 15–19 years), sex and type of injuries (per 100,000 population)	4.3	3.3	Unsure of data availability
10. Percentage of adolescents (10–19 years) experiencing contact sexual violence in the past 12 months, by age group (10–14 and 15–19 years), sex and perpetrator	4.3	2.9	Stigma
11. Percentage of young women and men (18–29 years) who experienced sexual violence by age 18, by age at victimisation ( < 10, 10–14 and 15–18 years), sex and perpetrator	4.2	2.7	Stigma
12. Percentage of adolescents (10–19 years) involved in bullying within the past 12 months, by type of involvement (victim, perpetrator and both), type of bullying (in person and digital/cyber), age group (10–14 and 15–19 years) and sex	4.8	3.4	Stigma
Mental health			
13. Percentage of adolescents (10–19 years) with depression and/or anxiety seeking mental healthcare or psychosocial support by age group (10–14 and 15–19 years) and sex	4.4	3.4	Wording of indicator
14. Percentage of adolescents (10–19 years) with depression and/or anxiety by age group (10–14 and 15–19 years) and sex	4.4	3.3	Wording of indicator
15. Percentage of adolescents (10–19 years) with a positive connection with their parent or guardian by age group (10–14 and 15–19 years) and sex	3.7	2.6	Hard to collect
16. Percentage of adolescents (10–19 years) reporting current (past 2 weeks) suicidal thoughts by age group (10–14 and 15–19 years) and sex	4.0	2.6	Stigma
17. Percentage of adolescents (10–19 years) with someone to talk to when they have a worry or problem by age group (10–14 and 15–19 years) and sex	4.0	3.2	Hard to collect

## 9 Not Relevant and Not Feasible Indicators

There were 11 out of 50 indicators that stakeholders perceived as irrelevant and not feasible to collect in the West Bank. These indicators involve alcohol, unchangeable cultural norms and unavailable practices and services. Table 6 describes all indicators found to be irrelevant and not feasible and why they were excluded.

**Table 6:** List of indicators irrelevant and not feasible indicators and the reason for exclusion.

Indicator name	Relevance	Feasible	Reason for exclusion
Determinants of health (education and income level)			
1. Percentage of adolescents (10–19 years) at the end of primary and at the end of lower secondary, achieving at least a minimum proficiency level in (i) reading and (ii) mathematics by age group (10–14 and 15–19 years) and sex	3.4	3.1	Not a priority
2. Percentage of adolescents (10–19 years) living below the international poverty line by age group (10–14 and 15–19 years) and sex	3.4	3.1	Not a priority
Sexual and reproductive health			
3. Percentage of adolescents (15–19 years) who had their first sexual intercourse before 15 years of age by sex	2.7	1.5	Unchangeable cultural norms
4. Percentage of female adolescents (15–19 years) who make their own informed decisions regarding sexual relations, contraceptive use and reproductive healthcare	3.1	2.3	Unchangeable cultural norms
5. Prevalence of contraceptive use (modern method) among adolescents (10–19 years) by age group (10–14 and 15–19 years), sex and method used	3.3	2.7	Unchangeable cultural norms
6. Percentage of adolescents (10–19 years) who have their need for contraception satisfied with modern methods by age group (10–14 and 15–19 years) and sex	3.2	2.4	Unchangeable cultural norms
7. Percentage of adolescents (10–19 years) who used a condom at last intercourse, by age group (10–14 and 15–19 years) and sex	2.3	1.6	Unchangeable cultural norms
8. Percentage of adolescents (15 years) covered by the HPV vaccine (last dose in schedule), by sex	2.2	2.0	Service not available
9. Percentage of female adolescents (10–19 years) who have undergone female genital mutilation/cutting by age group (10–14 and 15–19 years)	1.2	1.3	Not a burden
Health behaviour and risks			
10. Past 30‐day prevalence of heavy episodic drinking among adolescents (10–19 years), age group (10–14 and 15–19 years) and sex	2.9	1.9	Not a burden
11. Prevalence of current (past 30 days) alcohol use among adolescents (10–19 years) by age group (10–14 and 15–19 years) and sex	3.0	2.1	Not a burden

### 9.1 Alcohol Use

Alcohol was not considered a burden amongst adolescents. Access to alcohol is minimal. Furthermore, it is not feasible to collect data on use due to the topic being socially sensitive, as its use is forbidden in Islam. Alcohol behaviour questions are taken from school surveys in some Eastern Mediterranean countries [25]. In reviewing a survey with a stakeholder, alcohol behaviour questions were removed from the Global School Health Survey (GSHS) before being administered to the students in the West Bank.

### 9.2 Unchangeable Cultural Norms

Seven out of the 11 indicators fell under the topic of sexual reproductive health (SRH). Five indicators involved measuring contraception use and unmet needs, age of sexual intercourse, and condom use. Stakeholders felt that due to the increase of the age limit on marriage to 18 as of late 2021, they did not feel these issues would be a problem in the future. This shows that legal reform is important, but it overlooks other factors such as cultural resistance to the discussion about sexual health.

With the increase of the marriage age limit to 18, we will have fewer adolescent girls that need contraception, and it is unacceptable to ask questions about contraception and sexual intercourse to unmarried girls.

Also, these indicators are not acceptable to unmarried adolescents. The assumption is made because of Islamic and Christian views that sex before marriage is forbidden and that adolescents are not sexually active. These indicators are usually collected from school surveys, and married adolescents do not attend school. The exclusion of married adolescents from school‐based surveys creates a gap in the understanding of their health needs. It reinforces societal norms that marginalise this group, preventing the development of inclusive policies and programmes that address their sexual and reproductive health.

Once a girl gets married, she is not allowed to go back to school. This is because parents and teachers do not want married girls to describe their sexual relationships with the others in the classroom.

Along with unchangeable cultural norms, indicators were excluded due to unavailable services and practices.

## 10 Services/Practice Unavailable

Stakeholders found that it was not relevant and not feasible to collect indicators concerning the HPV vaccine and female genital mutilation. The HPV vaccine is not a service currently offered in the country, and stakeholders described that cervical cancer is also not prevalent. In addition, female genital mutilation is not practised, so they did not find a benefit in measuring this indicator. It is important to continuously assess the situation to ensure the assumption that HPV and female genital mutilation are not relevant to the local context.

## 11 Additional Indicators for Consideration

Most stakeholders expressed the need to include additional indicators not included in the original list, such as child labour, disability and electronic cigarettes. For example, they felt child labour and disability indicators should be included in the relevant and feasible list of indicators.

Many adolescents are leaving school and going to work in Israel, and we must measure this.

This highlights the growing concern over the socio‐economic factors affecting adolescents and their well‐being. It's important to have this data to address the concern of child labour. Although Palestine currently collects disability indicators, there was agreement that the current indicators were insufficient. This shows that stakeholders are aware of the gap in the current system of the indicators not properly capturing the prevalence, type or impact of disabilities on adolescents. Therefore, an agreement was made to explore the disability indicators with the proper stakeholders to decide which indicators should be included. It was also discussed that electronic cigarettes should be added to the tobacco indicator. Including this indicator is important because it shows that stakeholders are aware that there are newer forms of substance use that are becoming more prevalent, and it is important to monitor.

## 12 Opportunities and Challenges in the Creation of a National List of Adolescent Health Indicators

The first‐time stakeholders took the list of indicators into consideration: the need for adolescent health information and the need for measurement. All stakeholders expressed the need for the list to guide adolescent health programmes and policies.

This list is a great start in focusing on the need of adolescent health and evidence‐based programs.

This showed that stakeholders found that a standardised indicators list could play a huge role in evidence‐based decision‐making for adolescent health. The list has the potential to serve as an important catalyst for interventions and resource allocation.

Three main challenges were addressed by stakeholders in the workshop in creating a minimal list of indicators. One of the main challenges is the need for governmental support in the national list of indicators for adolescents. If there is government support for the list of indicators, it can help in leading the collection of indicators. Without a clear commitment from the government, the integration and sustainability of the list into the system are not feasible. A stakeholder described that many surveys are conducted based on funding availability instead of need.

Often, population‐based surveys are conducted because a funding body is interested in administering a certain survey. We must move away from funding‐driven surveys and focus on the necessary indicators.

This statement shows that external funding priorities often dictate data collection and not local needs. It is important to find a way to allow for donor priorities to align with local priorities. The other challenges described were who should pay for the data to be collected and what type of infrastructure would be needed to collect the data. Many of the stakeholders feel that the government should be in charge of taking the lead in owning the list of indicators and providing the information for said indicators. Exploring partnerships between government, international organisations and local stakeholders could be a way to address these challenges.

## 13 Discussion

Stakeholders identified 22 out of 50 GAMA adolescent health indicators as relevant and feasible. Seventeen indicators were considered relevant but not feasible with certain changes and can be feasible to collect in the country, and 11 were considered not relevant and not feasible, as they are very hard to collect in the West Bank. Still, the 17 indicators are considered relevant but not feasible. No indicator received a rating of higher than 4.5 in feasibility. Many of the included indicators had scores of 3.5 for feasibility. The unfeasible indicators came from the categorisation of sexual and reproductive health, violence and injury, and mental health.

Cultural and social norms were one of the main reasons some indicators were not feasible to collect in the West Bank. This is expected to be a problem in other countries in the Eastern Mediterranean Region. Sex before marriage is not socially acceptable to discuss. In a recent study in Saudi Arabia, women acknowledged their marriage status when discussing sexual and reproductive health topics [26]. Another study in Iran showed that university students initiated sexual intercourse after marriage. Still, a percentage did not have the information needed to perform safe sexual behaviours [27]. Girls also often know nothing about menstruation before it starts [28].

The Palestinian Educational System is currently working on incorporating sexual education, mental health, gender roles, and gender equity and equality into the educational curriculum. There is no sexual education programme in the current system. Furthermore, two studies have been done on the perception of sexual education and knowledge of university students in different geographical regions in the Occupied Palestinian Territory. Results showed that university students have insufficient knowledge of SRH rights, as well as limited awareness of the transmission of sexually transmitted infections and HIV/AIDS [29, 30]. A potential reason for the disparity in SRH knowledge was noted between students who attended private schools in the occupied Palestinian territory, where co‐educational settings and some sexual education might be provided [29, 30]. The lack of open discussion about SRH allows the formation of misconceptions, leaving adolescents and young adults without the tools to make informed decisions about their health.

Globally, comprehensive sexual education has been shown to improve SRH knowledge, reduce risky behaviours and promote gender equity. Knowledge of menstruation and puberty is also a part of comprehensive sexual education [31]. Yet, in culturally conservative settings like Palestine, introducing it into the curriculum requires a tactical approach. Community resistance, driven by fears that discussing sexual health may encourage premarital sexual activity, presents a significant barrier. There is a belief amongst parents and teachers that if sexual and reproductive health is discussed, sexual behaviours are more likely to increase. Studies have shown that comprehensive sexual education has not affected the desire for youth or adolescents to perform sexual behaviours. The educational curriculum has led to safer sexual behaviours but not increased sexual behaviours [32].

Some of the main programmes run in the country are mental health, violence and injuries, sexual and reproductive health and WASH. If it is not acceptable and a priority for these areas to be measured, then how are we measuring the success of our programmes? The main issues found in the mental health indicators were the wording of the indicator itself and the ability to collect the indicators. The ability to collect mental health indicators is a worldwide concern. Mental health data availability is scarce, particularly in low‐ and middle‐income countries, as over 100 countries were found to have no data, and Palestine is no exception [33]. The wording was found to be a problem because the focus of mental health in humanitarian settings is on post‐traumatic stress disorders and depression, as this can be misleading and subjective in disasters and armed conflict [34]. These indicators risk excluding other significant mental health challenges, such as anxiety, adjustment disorders or substance use. Also, the cultural differences in how distress is expressed emphasise the need for culturally adapted indicators. There is a need for the measurement of other mental health disorders or problems. This need was recognised with the formation of the MMAP initiative (Measurement of Mental Health among Adolescents at the Population Level). The initiative hopes to address the different mental health indicators in various situations [35]. By collaborating with MMAP, stakeholders in Palestine could refine mental health indicators to ensure cultural appropriateness, feasibility and representativeness.

Sexual violence and injuries were seen as unfeasible due to the stigma involved. Many times, sexual abuse of female children and adolescents goes unreported for fear of hurting the family reputation and future marriage prospects [36]. This is also a problem in the region. In a systematic review done on the key forms of violence against adolescents in the Arab region, it was found that the available data were insufficient in giving a clear picture of the prevalence of violence in the region. Many times, the sample sizes were small, showing that there were areas of violence where more research was needed [37]. Another review of child sexual abuse in Arab countries showed the under‐researched area as well. Many of the included studies showed cultural adaptation to questions to refrain from using sexual terms when describing sexual abuse. When the adaptations were not done, response rates were found to be lower than when adaptations were considered [38]. To address the stigma around sensitive health topics, implementing culturally adapted programmes and anonymous reporting systems may help provide an environment where adolescents feel safer sharing their experiences. Studies from similar humanitarian settings suggest that engaging community leaders and educators can help normalise discussions around mental health and sexual health, reducing stigma over time [39].

Stakeholders were open to adapting the relevant and feasible indicators, although many challenges were addressed. A comprehensive understanding of how data collection was operationalised, considering challenges and highlighting the significance of various factors like political will, leadership roles, costs, and responsibilities in this process, is needed. One of the main challenges faced in humanitarian crises is the collaboration of agencies and validation of data collection [40].

### 13.1 Strengths and Limitations

One of the main strengths is that this is the first time indicators were discussed and set in the West Bank for adolescent health. The topic being new will help lead towards the practice of measurement and evidence‐based programmes and policies. We contacted stakeholders with various backgrounds to fill in the ranking of indicators, ensuring that not one area of measurement was neglected. One of the limitations was the lack of generalisability of the results to the entire country. No stakeholder from the Gaza Strip was included in the ranking. Many were contacted, but with the inability of researchers to reach the Gaza Strip, they were not included in the study. Another limitation was the shortage of academics in the West Bank who specialise in adolescent health research. Only one academic was found, and the researchers conducting the study were not included.

## 14 Conclusion and Recommendations

Stakeholders agreed that 22 of the 50 adolescent health indicators were relevant and feasible to be collected in the West Bank. This does not mean that these should be the only indicators that should be measured and collected. The real challenge lies in the 17 indicators that were found to be relevant but not feasible. A way to overcome cultural norms would be to offer culturally sensitive, comprehensive sexual education. Comprehensive sexual education would be essential to be added to school curricula [41]. With an increase in the use of the internet and the vast variety of material available, comprehensive sexual education in schools allows teachers and counsellors to share correct and trusted information instead of adolescents looking for it themselves [42]. Cultural sensitivities should be considered, starting with awareness of gatekeepers such as religious leaders, teachers, counsellors and parents. The community's involvement will allow for change and acceptance of discussing sexual and reproductive health, allowing for the feasibility of these indicators to be collected [43]. It would also be important to change the wording of the mental health indicators to reflect humanitarian setting needs, as well as move away from the stigma involved with sexual abuse and suicidal thoughts by providing anonymous ways of reporting and speaking with professionals when needed. Implementing school‐based mental health programmes as well as antibullying programmes is the most efficient way to encourage the discussion of mental health in any country [44]. It is also important to find the resources to invest in the measurement of some of these indicators, as measuring will save resources in the future [45].

Considering the findings, small but impactful steps can be taken as the next course of action, as outlined below:

1. Developing pilot programmes for comprehensive sexual education that are tailored to local cultural and religious contexts. These programmes should be implemented in a small number of schools first, with thorough evaluation to address potential barriers and ensure scalability.
2. Developing and conducting comprehensive sexual education training for teachers, school nurses and counsellors that is culturally sensitive to the context, to allow educators to be more confident in providing this training.
3. Establishing anonymous reporting mechanisms for sensitive mental health issues, such as suicidal thoughts or sexual abuse. Digital platforms, helplines or secure reporting boxes in schools can be used, and educators can be trained to detect possible mental health issues.
4. Integrating mental health and anti‐bullying programmes into the school curriculum. Using interactive and participatory methods to normalise discussions around mental health to reduce stigma.
5. Develop a monitoring and evaluation (M&E) framework to track progress in implementing these recommendations. Include clear timelines, measurable outcomes and accountability mechanisms to ensure success.

These steps serve as a foundation for actionable change, ensuring that findings are effectively translated into meaningful improvements in adolescent health in the West Bank.

## 15 Contribution to the Field Statement

This study makes a significant contribution to the field of adolescent health in the Eastern Mediterranean region, particularly in humanitarian settings. It addresses the critical issue of overlooking adolescents' health needs during crises by identifying 22 relevant and feasible indicators to monitor their health in the West Bank. Work will be needed to help collect the 17 indicators deemed as relevant but not feasible. With limited data available on adolescent health in such contexts, this study fills an essential gap and advocates for evidence‐based programmes and policies. By engaging diverse stakeholders and advocating for culturally sensitive, comprehensive sexual education and mental health programmes, the study aims to overcome cultural and social barriers that hinder data collection on sensitive topics. The establishment of a national list of adolescent health indicators serves as a milestone for standardising data collection, guiding health programme planning and fostering accountability. Overall, this study's findings will significantly improve understanding, interventions and prioritisation of adolescent health in the region, potentially influencing similar contexts worldwide.

## Author Contributions

**Aisha Shalash:** conceptualisation, writing – original draft, methodology, writing – review and editing, data curation. **Niveen Abu Rmeileh:** supervision, funding acquisition, writing – review and editing, conceptualisation. **Dervla Kelly:** conceptualisation, writing – review and editing, supervision. **Khalifa Elmusharaf:** conceptualisation, data curation, writing – review and editing, supervision.

## Ethics Statement

The Institute of Community and Public Health, Birzeit University's ethical committee review board, has approved this study. Each participant was informed that their participation was voluntary and that, at any stage, they could withdraw their participation. They were also informed that their answers were not identifiable and that their participation would remain anonymous. No animal studies are presented in this manuscript. No potentially identifiable human images or data are presented in this study.

## Conflicts of Interest

The authors declare no conflicts of interest.

## Supporting information

Core Adolescent Health Indicators in Humanitarian Settings Annex 1.

## Data Availability

The authors will make the raw data supporting this article's conclusions available without undue reservation.
