# Supplementary material for: The Feasibility and Relevance of Collecting Adolescent Health Indicators in Humanitarian Settings: Results From the West Bank
Source: Health Expect. 2025 Jun 13;28(3):e70324. doi: 10.1111/hex.70324 (PMC12163940; doi:10.1111/hex.70324)
Supplement: Supplementary file 1 — Core Adolescent Health Indicators in Humanitarian Settings Annex 1. [file HEX-28-e70324-s001.docx]

Annex 1

**Core Adolescent Health Indicators in Humanitarian Settings: Palestine as a Case Study**

Background and Objectives

Identifying a core list of adolescent health indicators is part of a bigger project, “Strengthening of the Adolescent Health information System in Palestine.” In a prior activity, we accumulated all adolescent health indicators collected in Palestine and four other humanitarian countries. This included all indicators, definitions, sources of data to estimate these indicators, and age. Using these indicators and the list of core indicators recommended by Global Action for Measurement of Adolescent Health (GAMA) convened by the World Health Organization (WHO), we will make a list of core indicators found to be a priority in Palestine.

Methodology

We will interview key stakeholders involved in adolescent health. There is a list of 50 indicators and each participant will be asked on a scale of 1 to 5; 1 being not relevant to 5 being very relevant in consideration of each category for the ranking of each indicator, the following questions will be considered:

1. Why do you think this indicator is essential?

2. Why do you think the indicators that you did not select should not be core indicators?

1. Do you think we have the resources available to collect this indicator? Why or Why not?
2. How do you think the data for this indicator can be used?
3. What are some adolescent health indicators that should be included in the list of Core Indicators that are not currently f found?
4. How often do you think we can collect these indicators?

You can find the indicators and their translation on the next page.

| Name of Indicator | Context Specific | Feasibility of Measurement | Timely (Consistently Measurable) | Resources available (human +Financial) | Usefulness/ Important | Comments |
| --- | --- | --- | --- | --- | --- | --- |
| 1.Percentage of total population that are adolescents (10–19 years), by age category (10-14, 15-19 years) and sex  النسبة المئوية لمجموع السكان من المراهقين (10-19 سنة)، حسب الفئة العمرية (10-14 ، 15-19 سنة) والجنس |  |  |  |  |  |  |
| 2. Percentage of adolescents completing primary, lower secondary, and upper secondary school, by level and sex  النسبة المئوية للمراهقين الذين أتموا التعليم الابتدائي والإعدادي والثانوي ، حسب المستوى والجنس |  |  |  |  |  |  |
| 3. Percentage of adolescents (10-19 years) living below the national poverty line, by age group (10- 14, 15-19 years) and sex  النسبة المئوية للمراهقين (10-19 سنة) الذين يعيشون تحت خط الفقر الوطني ، حسب الفئة العمرية (10-15،14-19 سنة) والجنس |  |  |  |  |  |  |
| 4. Percentage of adolescents (10-19 years) living below the international poverty line, by age group (10-14, 15-19 years) and sex  النسبة المئوية للمراهقين (10-19 سنة) الذين يعيشون تحت خط الفقر الدولي ، حسب الفئة العمرية (10-14 ، 15-19 سنة) والجنس |  |  |  |  |  |  |
| 5. Percentage of adolescents (10-19 years) living with moderate or severe food insecurity in the population, based on the Food Insecurity Experience Scale (FIES), by age group (10-14, 15-19 years) and sex  النسبة المئوية للمراهقين (10-19 سنة) الذين يعيشون مع انعدام أمن غذائي معتدل أو شديد بين السكان ، بناءً على مقياس تجربة انعدام الأمن الغذائي(FIES) حسب الفئة العمرية (10-14 ، 15-19 سنة) والجنس |  |  |  |  |  |  |
| 6. Percentage of adolescents (10-19 years) not in education, employment, or training, by age group (10-14, 15-19 years) and sex  النسبة المئوية للمراهقين (10-19 سنة) غير الملتحقين بالتعليم أو العمل أو التدريب ، حسب الفئة العمرية (10-14 ، 15-19 سنة) والجنس |  |  |  |  |  |  |
| 7. Percentage of adolescents (10-19 years) at the end of primary; and at the end of lower secondary achieving at least a minimum proficiency level in (i) reading and (ii) mathematics, by age group (10-14, 15-19 years) and sex  سبة المراهقين (10-19 سنة) في نهاية المرحلة الابتدائية ؛ وفي نهاية المرحلة الإعدادية تحقيق ما لا يقل عن الحد الأدنى من مستوى الكفاءة في (1) القراءة و (2) الرياضيات ، حسب الفئة العمرية (10-14 ، 15-19 سنة) والجنس |  |  |  |  |  |  |
| 8.Percentage of female adolescents (15-19 years) who make their own informed decisions regarding sexual relations, contraceptive use and reproductive health care  النسبة المئوية للمراهقات (15-19 سنة) اللاتي يتخذن قراراتهن المستنيرة فيما يتعلق بالعلاقات الجنسية واستخدام موانع الحمل والرعاية الصحية الإنجابية |  |  |  |  |  |  |
| 9. Prevalence of overweight and obesity among adolescents (10-19 years), by weight status (overweight, obese), age group (10-14, 15-19 years), and sex  انتشار زيادة الوزن والسمنة بين المراهقين (10-19 سنة) ، حسب حالة الوزن (زيادة الوزن ، السمنة) ، الفئة العمرية (10-14 ، 15-19 سنة) ، والجنس |  |  |  |  |  |  |
| 10.Prevalence of thinness among adolescents (10-19 years), by age group (10-14, 15-19 years), and sex  انتشار النحافة بين المراهقين (10-19 سنة)، حسب الفئة العمرية (10-14 ، 15-19 سنة) والجنس |  |  |  |  |  |  |
| 11. Past 30 day prevalence of heavy episodic drinking among adolescents (10-19 years), age group (10-14, 15-19 years) and sex  معدل انتشار الإفراط في تناول الكحوليات بشكل عرضي خلال الثلاثين يومًا الماضية بين المراهقين (10-19 عاماً) ،الفئة العمرية (10-14 ، 15-19 عاماً) والجنس |  |  |  |  |  |  |
| 12. Past 12 month prevalence of psychoactive drug use among adolescents (10–19 years), by age group (10-14, 15-19 years), sex and by type of substances  معدل انتشار تعاطي المخدرات ذات التأثير النفساني خلال الاثني عشر شهرًا الماضية بين المراهقين (10-19 عامًا) ، حسب الفئة العمرية (10-14 ، 15-19 عامًا) ، الجنس ونوع المواد |  |  |  |  |  |  |
| 13. Prevalence of current (past 30 days) use of tobacco products among adolescents (10–19 years), by age group (10-14, 15-19 years), sex, and type of tobacco used  معدل انتشار الاستخدام الحالي (الثلاثين يوما الماضية) لمنتجات التبغ بين المراهقين (10-19 عاماً) ، حسب الفئة العمرية (10-14 ، 15-19 عاماً) ، الجنس ، ونوع التبغ المستخدم |  |  |  |  |  |  |
| 14. Percentage of adolescents (10–19 years) who consume at least 5 servings of fruit and vegetables daily, by age group (10-14, 15-19 years) and sex  النسبة المئوية للمراهقين (10-19 سنة) الذين يستهلكون على الأقل 5 حصص من الفاكهة والخضروات يومياً ، حسب الفئة العمرية (10-14 ، 15-19 عاماً) والجنس |  |  |  |  |  |  |
| 15. Percentage of adolescents (10–19 years) who have accumulated an average of at least 60 minutes per day of moderate‐vigorous physical activity in the previous week, by age group (10-14, 15-19 years)and sex  النسبة المئوية للمراهقين (10-19 عاماً) الذين تراكم لديهم ما لا يقل عن 60 دقيقة يومياً من النشاط البدني المعتدل و النشط في الأسبوع السابق ، حسب الفئة العمرية (10-14 ، 15-19 عاماً) والجنس |  |  |  |  |  |  |
| 16. Percentage of adolescents (10-19 years) involved in bullying within the past 12 months, by type of involvement (victim, perpetrator, both), type of bullying (in-person, digital/cyber), age group (10- 14, 15-19 years), and sex  النسبة المئوية للمراهقين (10-19 عاماً) المتورطين في التنمر خلال الـ 12 شهر الماضية ، حسب نوع المشاركة (الضحية ، الجاني ، كلاهما) ، نوع التنمر (شخصياً ، رقمي / إلكتروني) ، الفئة العمرية (10-14 ، 15-19 سنة) والجنس |  |  |  |  |  |  |
| 17. Percentage of adolescents (15-19 years) who had their first sexual intercourse before 15 years of age, by sex  النسبة المئوية للمراهقين (15-19 عاماً) الذين مارسوا أول اتصال جنسي لهم قبل سن 15 عاماً ، حسب الجنس |  |  |  |  |  |  |
| 18. Percentage of live births to female adolescents (10-19 years) attended by skilled health personnel, by age group (10-14, 15-19 years)  النسبة المئوية للمواليد الأحياء للمراهقات (10-19 سنة) تحت إشراف موظفين صحيين مهرة ، حسب الفئة العمرية (10-14 ، 15-19 سنة) |  |  |  |  |  |  |
| 19. Prevalence of contraceptive use (modern method) among adolescents (10-19 years), by age group (10-14, 15-19 years), sex and method used  انتشار استخدام وسائل منع الحمل (الطريقة الحديثة) بين المراهقين (10-19 سنة) ، حسب الفئة العمرية (10-14 ، 15-19 سنة) ، الجنس والطريقة المستخدمة |  |  |  |  |  |  |
| 20. Percentage of adolescents (10-19 years) who have their need for contraception satisfied with modern methods, by age group (10-14, 15-19 years) and sex  النسبة المئوية للمراهقين (10-19 سنة) الذين تقتنع حاجتهم إلى وسائل منع الحمل بالوسائل الحديثة ، حسب الفئة العمرية (10-14 ، 15-19 سنة) والجنس |  |  |  |  |  |  |
| 21. Prevalence of current (past 30 days) alcohol use among adolescents (10-19 years), by age group (10-14, 15-19 years) and sex  معدل انتشار تعاطي الكحول حالياً (آخر 30 يوماً) بين المراهقين (10-19 عاماً) ، حسب الفئة العمرية (10-14 ، 15-19 عاماً) والجنس |  |  |  |  |  |  |
| 22. Percentage of adolescents (10-19 years) who usually drank sugar-sweetened beverages once per day or more during the past 30 days, by age group (10-14, 15-19 years) and sex  النسبة المئوية للمراهقين (10-19 سنة) الذين عادة ما يشربون المشروبات المحلاة بالسكر مرة واحدة يومياً أو أكثر خلال الثلاثين يوماً الماضية ، حسب الفئة العمرية (10-14 ، 15-19 عاماً) والجنس |  |  |  |  |  |  |
| 23. Percentage of female adolescents (10-19 years) who were aware of menstruation before menarche, by age group (10-14, 15-19 years)  نسبة المراهقات (10-19 سنة) اللاتي كنَ على علم بالحيض قبل الحيض ، حسب الفئة العمرية (10-14 ، 15-19 سنة |  |  |  |  |  |  |
| 24. Existence of a functional adolescent (10-19 years) health program with coverage at the national level  وجود برنامج صحي فعال للمراهقين (10-19 سنة) برنامج صحي مع تغطية على المستوى الوطني |  |  |  |  |  |  |
| 25. Existence of national standards for the delivery of health services to adolescents (10-19 years)  وجود معايير وطنية لتقديم الخدمات الصحية للمراهقين (10-19 سنة) |  |  |  |  |  |  |
| 26. Existence of national policy exempting adolescents (10-19 years) from user fees for specified health services in the public sector, by type of service  وجود سياسة وطنية تعفي المراهقين (10-19 سنة) من رسوم الانتفاع بخدمات صحية محددة في القطاع العام ، حسب نوع الخدمة |  |  |  |  |  |  |
| 27. Existence of a legal age limit for married and unmarried adolescents (10-19 years) to provide consent, without spousal/parental/legal guardian consent, for specified adolescent health services, by marital status and type of service  وجود حد قانوني للسن للمراهقين المتزوجين وغير المتزوجين (10-19 عامًا) لتقديم الموافقة ، دون موافقة الزوج / الوالدين / الوصي القانوني ، للحصول على خدمات صحية محددة للمراهقين ، حسب الحالة الاجتماعية ونوع الخدمة |  |  |  |  |  |  |
| 28. Percentage of adolescents (10-19 years) using specified health services in the public or private sector within the past 12 months, by sector, age group (10-14, 15-19 years) and sex  النسبة المئوية للمراهقين (10-19 سنة) الذين يستخدمون خدمات صحية محددة في القطاع العام أو الخاص خلال الأشهر الـ 12 الماضية ، حسب القطاع والفئة العمرية (10-14 ، 15-19 سنة) والجنس |  |  |  |  |  |  |
| 29. Percentage of adolescents (15 years) covered by HPV vaccine (last dose in schedule), by sex  نسبة المراهقين (15 سنة) المشمولين بلقاح فيروس الورم الحليمي البشري (آخر جرعة في الجدول) ، حسب الجنس |  |  |  |  |  |  |
| 30. Existence of age‐ and sex‐disaggregated health data for adolescents (10-19 years) in the national health information system  وجود بيانات صحية مصنفة حسب العمر والجنس للمراهقين (10-19 سنة) في نظام المعلومات الصحية الوطني |  |  |  |  |  |  |
| 31. Existence of a nationally-defined minimum package of school-based health and nutrition services based on local health priorities  وجود الحد الأدنى من الحزمة المحددة وطنياً من خدمات الصحة والتغذية المدرسية على أساس الأولويات الصحية المحلية |  |  |  |  |  |  |
| 32. Percentage of schools that provided life skills-based HIV and sexuality education within the previous academic year  النسبة المئوية للمدارس التي قدمت تثقيفاً جنسياً و حول فيروس نقص المناعة البشرية القائم على المهارات الحياتية خلال العام الدراسي السابق |  |  |  |  |  |  |
| 33. Percentage of adolescents (10-19 years) with someone to talk to when they have a worry or problem, by age group (10-14, 15-19 years) and sex  النسبة المئوية للمراهقين (10-19 سنة) مع شخص ما للتحدث معه عندما يكون لديهم قلق أو مشكلة ، حسب الفئة العمرية (10-14 ، 15-19 سنة) والجنس |  |  |  |  |  |  |
| 34. Percentage of adolescents (10-19 years) with a positive connection with their parent or guardian, by age group (10-14, 15-19 years) and sex  النسبة المئوية للمراهقين (10-19 سنة) الذين تربطهم علاقة إيجابية بوالديهم أو الوصي عليهم ، حسب الفئة العمرية (10-14 ، 15-19 سنة) والجنس |  |  |  |  |  |  |
| 35. Adolescent (10-19 years) mortality rate, by age group (10-14, 15-19 years) and sex  معدل وفيات المراهقين (10-19 سنة) حسب الفئة العمرية (10-14 ، 15-19 سنة) والجنس |  |  |  |  |  |  |
| 36. Adolescent (10-19 years) mortality rate, by specified causes of death, age group (10-14, 15-19 years) and sex  معدل وفيات المراهقين (10-19 سنة) ، حسب أسباب الوفاة المحددة ، والفئة العمرية (10-14 ، 15-19 سنة) والجنس |  |  |  |  |  |  |
| 37. Number of new adolescent (10-19 years) HIV infections per 1,000 uninfected adolescent population, by age group (10-14, 15-19 years) and sex  عدد المراهقين الجدد (10-19 سنة) المصابين بفيروس نقص المناعة البشرية لكل 1000 من المراهقين غير المصابين ، حسب الفئة العمرية (10-14 ، 15-19 سنة) والجنس |  |  |  |  |  |  |
| 38. Percentage of adolescents (10-19 years) who used a condom at last intercourse, by age group (10-14,15-19 years) and sex  النسبة المئوية للمراهقين (10-19 سنة) الذين استخدموا الواقي الذكري في آخر جماع ، حسب الفئة العمرية (10-14 ، 15-19 سنة) والجنس |  |  |  |  |  |  |
| 39. Percentage of adolescents (10-19 years) reporting a suicide attempt in the past 12 months, by age group (10-14, 15-19 years) and sex  النسبة المئوية للمراهقين (10-19 عاماً) الذين أبلغوا عن محاولة انتحار خلال الاثنى عشر شهراً الماضية ، حسب الفئة العمرية (10-14 ، 15-19 عاماً) والجنس |  |  |  |  |  |  |
| 40. Percentage of adolescents (10-19 years) with depression and/or anxiety, by age group (10-14, 15-19 years) and sex  النسبة المئوية للمراهقين (10-19 سنة) المصابين بالاكتئاب و / أو القلق ، حسب الفئة العمرية (10-14 ، 15-19 سنة) والجنس |  |  |  |  |  |  |
| 41. Percentage of adolescents (10-19 years) with depression and/or anxiety seeking mental health care or psychosocial support, by age group (10-14,15-19 years) and sex  النسبة المئوية للمراهقين (10-19 عاماً) المصابين بالاكتئاب و / أو القلق الذين يسعون للحصول على رعاية الصحة العقلية أو الدعم النفسي والاجتماعي ، حسب الفئة العمرية (10-14 ، 15-19 عاماً) والجنس |  |  |  |  |  |  |
| 42. Incidence rate of specified types of injuries among adolescents (10–19 years), and by age category (10-14, 15-19 years), sex and type of injuries (per 100,000 population)  معدل حدوث أنواع محددة من الإصابات بين المراهقين (10-19 سنة) ، وحسب الفئة العمرية (10-14 ، 15-19 سنة) ، الجنس ونوع الإصابات (لكل 100،000 من السكان) |  |  |  |  |  |  |
| 43. Percentage of adolescents (10-19 years) involved in physical violence in the past 12 months, by type of involvement (victim, perpetrator, both), age group (10-14, 15-19 years), sex, perpetrator (parents/caregivers, teachers, intimate partners, peers)  النسبة المئوية للمراهقين (10-19 سنة) المتورطين في العنف الجسدي خلال الـ 12 شهراً الماضية ، حسب نوع المشاركة (الضحية ، الجاني ، كلاهما) ، الفئة العمرية (10-14 ، 15-19 سنة) ، الجنس ، الجاني (الوالدان / مقدمي الرعاية والمعلمين والشركاء الحميمين والأقران) |  |  |  |  |  |  |
| 44. Percentage of adolescents (10-19 years) experiencing contact sexual violence in the past 12 months, by age group (10-14, 15-19 years), sex, and perpetrator  النسبة المئوية للمراهقين (10-19 عاماً) الذين عانوا من العنف الجنسي أثناء الاتصال خلال الاثني عشر شهراً الماضية ، حسب الفئة العمرية (10-14 ، 15-19 عاماً) ،الجنس والجاني |  |  |  |  |  |  |
| 45. Percentage of young women and men (18-29 years) who experienced sexual violence by age 18, by age at victimization (<10, 10-14, 15-18 years), sex, and perpetrator  النسبة المئوية للشباب والشابات (18-29 عاماً) الذين تعرضوا للعنف الجنسي حسب سن 18 سنة، حسب العمر عند الإيذاء (أقل من 10 ، 10-14 ، 15-18 عاماً) ،الجنس والجاني |  |  |  |  |  |  |
| 46. Adolescent (10-19 years) fertility rate, by age group (10-14, 15-19 years)  معدل خصوبة المراهقين (10-19 سنة) حسب الفئة العمرية (10-14 ، 15-19 سنة) |  |  |  |  |  |  |
| 47. The incidence rate of sexually transmitted infections (STIs) among adolescents (10-19 years), by age group (10-14, 15-19 years) and sex  معدل الإصابة بالأمراض المنقولة جنسياً بين المراهقين (10-19 سنة) حسب الفئة العمرية (10-14 ، 15-19 سنة) والجنس |  |  |  |  |  |  |
| 48. Percentage of adolescents (10-19 years) reporting current (past two weeks) suicidal thoughts, by age group (10-14, 15-19 years) and sex  النسبة المئوية للمراهقين (10-19 سنة) الذين أبلغوا عن أفكار انتحارية حالية (الأسبوعين الماضيين) ، حسب الفئة العمرية (10-14 ، 15-19 سنة) والجنس |  |  |  |  |  |  |
| 49. Percentage of female adolescents (10-19 years) who have undergone female genital mutilation/cutting, by age group (10-14, 15-19 years)  النسبة المئوية للمراهقات (10-19 سنة) اللاتي خضعن لتشويه / بتر الأعضاء التناسلية للأنثى ، حسب الفئة العمرية (10-14 ، 15-19 سنة) |  |  |  |  |  |  |
| 50. Prevalence of anemia among adolescents (10-19 years), by age category (10-14, 15-19 years) and sex  معدل انتشار فقر الدم بين المراهقين (10-19 سنة) حسب الفئة العمرية (10-14 ، 15-19 سنة) والجنس |  |  |  |  |  |  |
